# Improving the Health of Individuals With Cerebral Palsy: Protocol for the Multidisciplinary Research Program MOVING ON WITH CP

**DOI:** 10.2196/13883

**Published:** 2019-10-09

**Authors:** Ann Alriksson-Schmidt, Johan Jarl, Elisabet Rodby-Bousquet, Annika Lundkvist Josenby, Lena Westbom, Kate Himmelmann, Kristine Stadskleiv, Pia Ödman, Ingrid Svensson, Christian Antfolk, Nebojsa Malesevic, Ira Jeglinsky, Sanjib Saha, Gunnar Hägglund

**Affiliations:** 1 Department of Orthopedics Faculty of Medicine Lund University Lund Sweden; 2 Department of Health Economics Faculty of Medicine Lund University Lund Sweden; 3 Centre for Clinical Research, Västmanland-Uppsala University Västerås Sweden; 4 Department of Health Sciences Faculty of Medicine Lund University Lund Sweden; 5 Children’s Hospital Skåne University Hospital Lund Sweden; 6 Department of Paediatrics Faculty of Medicine Lund University Lund Sweden; 7 Department of Pediatrics Institute of Clinical Sciences Sahlgrenska Academy at the University of Gothenburg Gothenburg Sweden; 8 Department of Clinical Neurosciences for Children Oslo University Hospital Oslo Sweden; 9 Department of Medical and Health Sciences Faculty of Medicine Linköping University Linköping Sweden; 10 Department of Biomedical Engineering Lund University Lund Sweden; 11 Department of Health and Welfare Arcada University of Applied Sciences Helsinki Finland

**Keywords:** cerebral palsy, health care, pain, health, disability, multidisciplinary

## Abstract

**Background:**

Cerebral palsy (CP) is one of the most common early onset disabilities globally. The causative brain damage in CP is nonprogressive, yet secondary conditions develop and worsen over time. Individuals with CP in Sweden and most of the Nordic countries are systematically followed in the national registry and follow-up program entitled the Cerebral Palsy Follow-Up Program (CPUP). CPUP has improved certain aspects of health care for individuals with CP and strengthened collaboration among professionals. However, there are still issues to resolve regarding health care for this specific population.

**Objective:**

The overall objectives of the research program MOVING ON WITH CP are to (1) improve the health care processes and delivery models; (2) develop, implement, and evaluate real-life solutions for Swedish health care provision; and (3) evaluate existing health care and social insurance benefit programs and processes in the context of CP.

**Methods:**

MOVING ON WITH CP comprises 9 projects within 3 themes. Evaluation of Existing Health Care (Theme A) consists of registry studies where data from CPUP will be merged with national official health databases, complemented by survey and interview data. In Equality in Health Care and Social Insurance (Theme B), mixed methods studies and registry studies will be complemented with focus group interviews to inform the development of new processes to apply for benefits. In New Solutions and Processes in Health Care Provision (Theme C), an eHealth (electronic health) procedure will be developed and tested to facilitate access to specialized health care, and equipment that improves the assessment of movement activity in individuals with CP will be developed.

**Results:**

The individual projects are currently being planned and will begin shortly. Feedback from users has been integrated. Ethics board approvals have been obtained.

**Conclusions:**

In this 6-year multidisciplinary program, professionals from the fields of medicine, social sciences, health sciences, and engineering, in collaboration with individuals with CP and their families, will evaluate existing health care, create conditions for a more equal health care, and develop new technologies to improve the health care management of people with CP.

**International Registered Report Identifier (IRRID):**

DERR1-10.2196/13883

## Introduction

### Background

All children should have the right to health and quality of life [1], yet children with disabilities have impairments, putting them at risk for reduced participation, limited involvement in everyday activities, and reduced quality of life [2,3]. Cerebral palsy (CP) is one of the most common early onset disabilities with a prevalence of 2 to 3 per 1000 live births [4,5]. Males are marginally overrepresented but there are no known sex differences in gross motor function [6,7]. The brain damage that causes CP is nonprogressive, but associated secondary conditions, by definition preventable [8], develop and deteriorate over time. Levels of function and types of comorbidities and secondary conditions vary greatly, and physical and occupational therapy, orthoses, medications, and a range of orthopedic surgeries are used to maximize physical function.

Since 1994, children and adolescents with CP in the southern regions of Sweden have been systematically enrolled in a follow-up program called the Cerebral Palsy Follow-Up Program (CPUP). By 2007, more than 95% of all youths with CP born in 2000 or later in all health care regions of Sweden were enrolled, and the program started receiving government funding and became a national quality registry. CPUP or adapted versions of it have been implemented in Norway, Denmark, Iceland, Scotland, Jordan, and New South Wales in Australia [9]. Data are systematically collected on variables such as gross motor function, mobility, hand function, range of motion, degree of spasticity, hip displacement, pain, and scoliosis (see www.cpup.se for a complete list). More recently, adults with CP are eligible to participate; however, the adult cohort in CPUP does not comprise the total population.

CPUP has improved certain aspects of health care for individuals with CP and strengthened collaboration among professionals. For instance, the prevalence of hip dislocations in children with CP was reduced from approximately 10% to 0.5% in a time span of 10 years, and the number of orthopedic surgeries has been reduced [10,11]. However, there are still numerous health care–related issues to resolve. Taxpayer-funded universal health care should be evidence-based, or based on best practice if scientific evidence is lacking, and provided equitably to those who need it, yet educational level, geographical residence, sex, age, and country of origin are factors known to influence access to health care [12-14]. Research has pointed to sex differences in the treatment of children with CP; boys are more likely to receive botulinum toxin A [15] and undergo selective dorsal rhizotomy [16]. In northern Sweden, boys with CP have been reported to receive more physical therapy than girls [17]. The concepts of equality (ie, everybody receives the same, regardless of individual needs and outcomes) and equity (ie, fairness and equality in outcomes is of main interest, meaning not everybody receives the same) need to be considered in the context of treatment. Sex differences in treatment might be warranted if, for instance, biological differences between the sexes (eg, boys might hypothetically be more spastic) exist. We have not found support for such a hypothesis. If differences in treatment exist, it is important to know why and what the consequences are.

Approaches to address CP-related health care differ. In CPUP, the focus is on prevention. One goal, for example, is to prevent hip dislocations before they occur by monitoring and following the children over time. For a preventive approach to be successful, it is necessary to be able to predict who is at risk and in need of preventive treatment. As an example, CPUP data have been used to develop risk scores to predict hip displacement [18]. People who for various reasons do not stretch their muscles sufficiently during the day are at risk of developing contractures [19]. It would, therefore, be useful to be able to monitor the movement and range of joint motion pattern during longer periods to predict the risk of contracture development. In other countries (eg, Finland), a reactive health care approach is used instead, and surgery is performed after the hip dislocations have occurred.

To enable prevention-focused work requires early detection and receiving an accurate CP diagnosis. According to the CPUP guidelines, a pediatric neurologist or specialist with similar expertise should confirm or dismiss the CP diagnosis by the time the child is aged 4 to 5 years. A shortage of certain specialists, including pediatric neurologists, is a challenge in Sweden. The number of pediatric neurologists employed in the habilitation setting, where children with CP receive much of their health care, has decreased, while the workload has increased. Furthermore, the number of children in Sweden with a confirmed or dismissed diagnosis of CP, per current guidelines, has decreased. Currently, 30% of those old enough to have a confirmed diagnosis of CP reported in CPUP have not yet been diagnosed [20]. Furthermore, sparsely populated rural regions are often less attractive to professionals, and such regions might be prone to a chronic lack of professionals and specialists. Better use and implementation of existing technology is one possible solution to this. Videoconferencing, online examinations, and online prescription refills are commonplace. In the context of CP, it is the process and implementation that need to be developed and evaluated, not the technology, per se. Engaging experienced pediatric neurologists long distance in diagnosing and treating individuals with CP within the CPUP framework is one possibility that will be explored and studied in this research program.

Pain is present already in young children with CP and increases with age [21-25], and researchers have cautioned that the pain is frequently not identified or that it is undertreated [23,24]. In spite of the decrease in hip dislocations [10], many who do not have dislocations still report pain in the hips [26]. One of the next challenges in Sweden is to develop a cost-effective pain prevention, reduction, and treatment program and implement it using the existing CPUP infrastructure. This requires knowledge of the pain panorama in individuals with CP.

Individuals with CP are at risk of experiencing cognitive difficulties. Although the degree of motor and cognitive impairment do correlate [27], there is no absolute correlation between motor and cognitive functioning [28,29]. Cognitive impairment can be found across the spectrum of severity of gross motor function, and it is warranted to assess all children with CP [30] and, arguably, adults. Nevertheless, cognition is often not assessed in this population [31] because of an assumption that children with CP cannot be cognitively assessed because of speech and motor impairments. However, research has shown that it is possible to assess 62% of children with CP with psychological tests in a standardized manner [29] and at least 80% if the mode of responding to the test questions is adapted (eg, pointing with eye-gaze instead of with a finger). Results from a cognitive assessment might indicate that assistive technologies are needed, that there is a need for augmentative and alternative communication (AAC), schools and work places can be informed in a timely manner, and appropriate expectations can be set. In addition, cognitive impairment needs to be considered when applying for social insurance and disability benefits.

In Sweden, children and youths with CP and certain other disabilities receive health care and treatment at habilitation centers free of charge. The multidisciplinary professionals at these centers are tasked with overseeing the overall health and development and providing the necessary health care for children with disabilities. Physicians working in habilitation centers spend a lot of time documenting and writing certificates to the Social Insurance Agency and local municipalities. These certificates serve as the basis for decisions regarding what benefits the children and their families are entitled to, how often, and how much. How social benefit claims are handled and decisions are made are burning political issues in Sweden. Many users with CP consider the benefit application haphazard and believe decisions are made based on geography, which physician completed the certificate, and who processed the claim.

### Overall Purpose and Specific Aims

**Figure 1:**
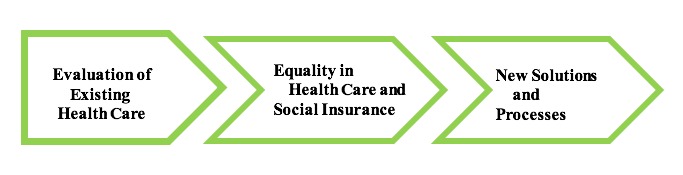
Research themes in MOVING ON WITH CP.

The objectives of the research program MOVING ON WITH CP are to (1) improve the health care processes and delivery models and thereby improve health, quality of life, and social participation in this population; (2) develop, implement, and evaluate real-life solutions for Swedish health care provision; and (3) evaluate existing health care and social insurance benefit programs and processes in the context of CP (Figure 1).

The specific aims are as follows:

- Evaluate the treatment effect, clinically and cost-effectively, of a population-based preventive approach (CPUP) compared to a reactive health care (regular care) approach (project A1)
- Investigate the prevalence, intensity, and specific body sites affected by pain and if this pain is related to demographic, socioeconomic, and disability factors (project A2)
- Investigate effects of pain on short- and long-term outcomes in relation to sleep, social participation, and labor market outcomes (project A2)
- Evaluate the implementation of the CPCognition (CPCog) protocol in Sweden and Norway (project A3)
- Determine what is relevant to study and report for adults with CP based on the International Classification of Functioning, Disability, and Health categories specific to CP (project A4)
- Study the effects of living with CP in terms of social participation and health and if the Swedish social insurance systems contribute to financial protection and enable acceptable living conditions (project B1)
- Determine if consequences of CP and access to formal societal support are equitably distributed based on disability and functional factors or if demographic and socioeconomic factors are related to access to societal support (projects B2 and B3)
- Develop, test, and evaluate a template based on structured and valid information on body functions from CPUP to be used by physicians in the certificate-writing process (project B3)
- Develop, test, and evaluate a device that continuously measures movement and range of joint motion (project C1)
- Develop, test, and evaluate an eHealth (electronic health) consultation service by tertiary care specialists using the infrastructures available through CPUP and MMCUP—the equivalent of CPUP but for individuals with a diagnosis of spina bifida (project C2)

## Methods

### Procedure and Data Sources

Summaries of the individual projects included in the program are presented in Multimedia Appendix 1 according to the three themes. Both quantitative and qualitative designs are included. The registry studies will be based on secondary analyses of preexisting data available in CPUP and general national registers (Statistics Sweden and the National Board of Health and Welfare). The data will be managed, analyzed, presented, and archived in compliance with national regulations, European Union directives (when applicable), Good Epidemiological Practice, and the requirements posed by the ethical review boards.

### Ethical Considerations

The current research program consists of a number of different projects. For the research projects that are based on CPUP data, ethical approvals are already in place (LU 443-99, revised 2009, and LU EPN 2017/78). Additionally, ethics approvals for the Finnish cohort data (project A1: HUS/3640/2017, project C1: 2019-02452, and project A4: MEC-2018-1126) are in place. We are in the process of obtaining ethical approval for the remaining projects. No research will commence until the required approvals have been obtained.

## Results

This is a protocol for a research program and there are no results to report to date. The program is funded by the Swedish Research Council for Health, Working Life, and Welfare. The CPUP User Board, which consists of individuals with CP and family members, has reviewed and provided feedback on the individual studies. The first results are expected to be available at the end of 2019, and scientific publications are expected to be forthcoming at the beginning of 2020.

## Discussion

### Summary

CP-related research has advanced during the last decades. Although we know more about the disability, there are numerous knowledge gaps and a number of health care delivery processes that could be improved. In MOVING ON WITH CP, we are addressing some of these knowledge gaps. Importantly, the program is multidisciplinary, and the research team consists of physicians, psychologists, public health scientists, physical therapists, health economists, and engineers. For research in general and applied research in particular, it is crucial to get the insights of those who are ultimately affected. In our case, that means individuals with CP and their families. By involving the CPUP User Board, we will ensure that the research we do and plan to do is actually relevant to the stakeholders. Moreover, the stakeholders can facilitate the knowledge brokerage by ensuring that the information to be communicated outside of the professional community is relevant and appropriate.

Research on disability can at times be hampered by small sample sizes. By using register-based research, it is possible to increase the statistical power while at the same time increasing the external generalizability and reducing selection bias. In CPUP, we have systematically followed individuals with CP for over two decades and we have longitudinal data available that enable us to evaluate a number of aspects in terms of health care and health care delivery. Moreover, through CPUP we also have the infrastructure in place to implement our findings as they become available. Contributing to the knowledge base is critical; however, an important purpose of MOVING ON WITH CP is to ensure that the health care of individuals with CP is evidence-based, up to date, cost effective, and fair. The type of health care received should not depend on a person’s geographical location or sex.

In order to move health care management of CP forward, we must use the development that takes place in technology and collaborate with experts in these areas. We will collaborate with technical experts to create an organization that compensates for inequalities in access to health care and create technical equipment that improves the assessment of movement activity in people with CP. To implement the purpose of MOVING ON WITH CP, three themes have been developed and encompass all projects.

### Limitations

There will be numerous limitations to the research program. However, these will be discussed in more detail as the results from the individual projects are reported.

### Conclusion

The results of this research program will be of interest to many different research areas such as medicine, social sciences, political science, health economics, as well as the fields of inequality, disability, and biotech research. More importantly, the results will lead to tangible improvements in Swedish health care. The findings will be disseminated to a wide audience including stakeholders (individuals with CP, families, and governmental agencies); the general public; user organizations; higher education students; policy makers; health care administrators/providers; and the scientific community.

## Appendix

Multimedia Appendix 1 Description of the studies in MOVING ON WITH CP.

Multimedia Appendix 2 Peer-reviewer report from Forte.

## Abbreviations

CP: cerebral palsy
CPCOG: Cerebral Palsy Cognition Module included in CPUP
CPUP: Cerebral Palsy Follow-Up Program
eHealth: electronic health
GMFCS: Gross Motor Function Classification System
ICF: International Classification of Functioning, Disability, and Health
MACS: Motor Ability Classification System
MMC: myelomeningocele
MMCUP: Myelomeningocele Follow-Up Program
