# Supplementary material for: Improving the Health of Individuals With Cerebral Palsy: Protocol for the Multidisciplinary Research Program MOVING ON WITH CP
Source: JMIR Res Protoc. 2019 Oct 9;8(10):e13883. doi: 10.2196/13883 (PMC6811769; doi:10.2196/13883)
Supplement: Multimedia Appendix 1 [file resprot_v8i10e13883_app1.pdf]

Multimedia Appendix 1. **Description of the studies in *MOVING-ON WITH CP***

Study title	Design	Participants/Data source and Methods
		**Theme A. Evaluation of Disability-Related Healthcare**
A1. Comparison of treatment effects of preventive and reactive approaches of habilitation practices, pain, prevalence of hip dislocations and scoliosis, and health economic outcomes.	Cohort studies with retrospective longitudinal register data and simulation modeling.<br><br>Survey and Qualitative study	In Finland (reactive care), a regional cohort of children/youth with CP will be used. Data from Sweden (preventive care) will be extracted from the CPUP national database. Hip/spine radiographs, the hip/scoliosis surgeries, and costs associated with the different healthcare approaches will be compared in a Markov model [32].<br><br>The Pain and Comfort Questionnaire [33] will be used to analyze pain and comfort. Experience of pain in relation to activity and participation will be explored by interviews. Descriptive statistics, ANOVA (survey) and manifest and latent content analysis (qualitative study) [34].
A2. Pain in adults with cerebral palsy.	Survey and cross-sectional studies.	Approximately 1,600 adults followed in CPUP. Variables to be included: Demographic (age, sex), socioeconomic (education, household income, migration status), and disability specific (gross motor function, communication and manual ability) variables. Effects of pain on sleep, social participation, and labor market outcomes will be studied. Data from CPUP will be merged with data from Swedish national registers (e.g.,

		Social Insurance Agency). Descriptive statistics, ANOVAs, and logistic regressions with interactions between key covariates. Effect of pain will be studied controlling for potential selection effects using propensity score approaches such as inverse-probability weighting. Fixed effect models will also be included.
A3. Evaluation of the implementation process of the ‘CPCog’ protocol in Sweden and Norway	Survey- and cross-sectional studies.	Cognition data from the national CP registries in Sweden and Norway will be supplemented with survey data to be filled out by parents of children with CP born between 2005 and 2013. There will also be a survey of how the professionals evaluate the protocol, and questionnaires will be sent to all habilitation units in both countries, to be answered by the psychologists and the leaders separately. Descriptive statistics, independent samples t-tests, chi-squares and Fischer’s exact tests will be used. Correlations and standard regression methods will be used to explore the relationship between *CPCog* and other variables and the defined outcomes, including interaction effects.
A4. Development of an International Classification of Functioning, Disability and Health (ICF) core	Empirical cross-sectional study based on interviews with	ICF categories specific for CP will be developed to specify what is relevant to study and report for adults with CP. The first step is a preparatory phase to collect scientific evidence from interviews with adults with CP in three different countries and an expert survey (health professional‘s perspective) then linking the results to the ICF categories. After this process, the most relevant ICF categories will be collected. The second step

set for adults with CP.	professionals and adults with CP	is an international consensus meeting by experts and health professionals to decide which categories to include in the final ICF Core Set. The last step is the implementation of the first version of the ICF Core Set in the healthcare setting.

Theme B. Equality in Healthcare and Social Insurance Programs

B1. Equality in healthcare and social insurance programs for persons living with CP	Longitudinal population-based register studies.	Individuals with CP and their parents will be compared to a matched control group and followed from 1991 to 2015. Information from Statistics Sweden (e.g., education, income, benefits), National Board of Health and Welfare (e.g., healthcare utilization), and national registers for CP/MMC (e.g., factors related to disability and function) will be linked to both the individuals and their caregivers.<br>Advanced statistical methods will be applied for example selection models (when factors affecting the likelihood of access are different from the factors that affect level of use), fixed effect estimations, and potential outcome models using propensity score (when factors are correlated to both risk of disability and specific outcomes).
B2. Evaluation of sex differences in CP-related treatments and interventions for	Cross-sectional retrospective registry study.	Pre-existing CPUP data from the latest physical therapy (n = 2,695) and occupational therapy (n = 3,480) forms from the years 2016-2017 will be extracted for children and adolescents 0-17 years-of-age.<br>Types and frequencies of treatments and interventions used to treat children CP will be analyzed using descriptive statistics.

boys and girls with CP.		Logistic regressions will be used to assess the relationships between the outcome variables and the place of birth adjusted for age, Gross Motor Function Classification System levels and Manual Ability Classification System
B3. *Development of improved, fair, and labor saving health insurance certificates*		This project consists of four phases: Phase I- focus groups with physicians, users, representatives from the Social Insurance Agency to establish what factors should determine access to benefits. Phase II- development of template, process, and infrastructure for writing certificates; Phase III- testing in pilot project and evaluation of the usefullness and cost-effectiveness; Phase IV- Implementation including short- and long term evaluation.
**Theme C. New solutions and Processes in Healthcare Provision**		
C1. Development of a home-based device to measure movement and range of motion.	Product development followed by cross-sectional and longitudinal clinical studies.	A wearable device, capable of measuring range of joint motion will be developed, tested, and evaluated. The device should be able to log data for > 24 hours. Firstly, several approaches to the technical design will be evaluated in a lab setting. The device will be extended to include electro myography amplifier/ digitizer to enable synchronous tracking of the child's muscular activities. The device will then be tested on a control group, to get baseline data and ensure proper longterm function. Significant effort will be made to minimize the dimensions of the rigid components and ensure easy and robust

		positioning of the device. Following a successful test, it will be tested on a group of children with CP.
C2. e-Health tertiary care services for diagnosing and treatment recommendations of individuals with rare disabilities.	Population based observational longitudinal study.	The population includes all CPUP participants, 5-14 years old, living in Sweden. Inventories at baseline and follow-up 12 months after start of the consultation service regarding diagnostic accuracy, and with identification of need for evaluation at a tertiary care intervention. An economic evaluation to establish the cost-effectiveness of the e-health tertiary care services will also be performed from both healthcare and social care perspective comparing to standard services from the trial duration.
