# Supplementary material for: Improving the Health of Individuals With Cerebral Palsy: Protocol for the Multidisciplinary Research Program MOVING ON WITH CP
Source: JMIR Res Protoc. 2019 Oct 9;8(10):e13883. doi: 10.2196/13883 (PMC6811769; doi:10.2196/13883)
Supplement: Multimedia Appendix 2 [file resprot_v8i10e13883_app2.pdf]

FORTE: Forskningsrådet för hälsa, arbetsliv och välfärd

Beredningsgruppens yttrande

2018-01468	Gunnar Hägglund	Beredningsgrupp: Vård18

**Utlysningsnamn:** Vårdforskning 2018	**Bidragsform:** Programbidrag
**Projekttitel (svenska):** FRAMÅT MED CEREBRAL PARES	**Sökt inriktning:** Vård

Inledning

En kort inledning som beskriver syftet med projektet

The overall purpose of MOVING-ON WITH CP is to improve the healthcare processes and the delivery models for individuals with early-onset, life-long disabilities, with a focus on Cerebral Palsy, and thereby improve health, QoL, and social participation in this population. The aim is for these processes and models to be cost-effective, and distributed equally across Sweden. We will develop, implement, and evaluate real-life solutions for Swedish healthcare provision. The program also involves evaluations of existing healthcare and social insurance programs and processes, with focus on inequality/health disparities. The program is divided into 3 overarching themes: Evaluation of disability-related healthcare; Equality in healthcare and social insurance programs; and New solutions and processes in healthcare provision.

Bedömning

Forskningsprogrammets vetenskapliga kvalitet (originalitet, teorianknytning samt relation till tidigare forskning inom området och den internationella forskningsfronten)

The program includes 8 projects that are linked, well-supported in the background section, and built nicely on previous research. The program is challenging, fills important research gaps, and is truly interdisciplinary.

Studiedesign, metoder och datamaterial

The methods of the 8 sub-projects are adequate and valid.
This program will use population-based longitudinal data. Sweden has a long tradition of well-kept national registers and coupled with the unique personal identification numbers, it is possible to merge data from many different types of registers, enabling research that is more comprehensive. Using these databases is excellent for answering the research questions.

Programmets vetenskapliga värde

Early detection, receiving an accurate diagnosis, and preventing secondary complications are key for appropriate treatment and maximizing QoL for this population.
Research on CP is generally based on convenience or clinic-based samples, which reduces generalizability. This program will use population-based longitudinal data on children and youth with CP in Sweden, which reduces the selection bias. Producing evidence-based cost-effective solutions to better care for this vulnerable population is a high priority.

Resultatens förväntade praktiska betydelse/relevans

Highly relevant for clinical practice and implementation opportunities seem excellent due to the excellent network and status of the group.

Samhällsrelevans

Improving care and reducing pain for people living with disabilities is relevant to society. Inclusion of cost-effectiveness study is also relevant to society given the limited resources available.

Genus- och mångfaldsperspektiv i forskningens innehåll

OK with regard to gender and SES.

Programmets genomförbarhet

Seems feasible.

Programledarens och forskargruppens forskningskompetens

PI has excellent track record and ample expertise in the field and in leadership. The group seems truly interdisciplinary and a strong consortium.

Personal och budget

OK

Sammanfattande bedömning

This is an excellent proposal with valid methods, high clinical relevance and optimal opportunities for implementation.

Etikprövning

Bedöms sakna etisk problematik eller behov av etikprövning finns

Approval needed.

Förslag till beslut

Bevilja, bevilja i mån av medel eller avslå

Approve
